# Foreign aid and economic growth: Do energy consumption, trade openness and CO_2_ emissions matter? A DSUR heterogeneous evidence from Africa’s trading blocs

**DOI:** 10.1371/journal.pone.0253457

**Published:** 2021-06-25

**Authors:** Yao Hongxing, Olivier Joseph Abban, Alex Dankyi Boadi

**Affiliations:** 1 Institute of Applied Systems and Analysis (IASA), School of Mathematical Science, Jiangsu University, Zhenjiang, P. R China; 2 School of Finance and Economics, Jiangsu University, Zhenjiang, P. R China; 3 Innovation and Consultancy, University of Cape Coast Directorate of Research, Cape Coast, Ghana; Institute for Advanced Sustainability Studies, GERMANY

## Abstract

The paramount vision of every country or sub-regions is to attain economic growth and sustainable economic growth. The paradigm drift of studies into foreign aid and sustainable economic growth has shown conflicting results that play on researchers to fill the gap of knowledge void. The plurality of studies looked at economic growth and foreign aid in single countries. However, one of the major determinants of sustainable growth such as CO_2_ emissions and trade goes beyond the boundaries of a country. Deductively, grouped countries or sub-regional studies are needed to ascertain the heterogeneous relationship and cross-sectional dependency among panels grouping. We fill these gaps with the recent empirical methodology to unveil the impact of foreign aid, CO_2_ emissions, trade openness, and energy consumption on economic growth. Thus a percentage rise in foreign aid corresponds to different significant weights in all panel groupings with exception of Southern African Development Community, which unveiled a non-significant estimate. Whereas trade openness in all panel grouping indicated a significant weight on economic growth. An increase in CO_2_ emissions has a significant material effect on economic growth in Common Market for Eastern and Southern Africa, Economic Community of West African States, and Community of Sahel-Saharan States. The impact of energy consumption on economic growth across the panel groupings was statistically significant with Common Market for Eastern and Southern Africa having the highest weight impact. These results obtained in this study indicate that foreign aid, energy consumption, trade openness, and CO_2_ emissions are positively correlated with economic growth. Based on the finding, the significant of the policy implications suggested. (a) The need for a paradigm shift from fossil fuel sources to renewables is encouraged in the various trading blocs (b) The need to embrace carbon storage and capturing techniques to decouple pollutant emissions from economic growth on the continent’s growth trajectory.

## 1. Introduction

Economic growth (per capita GDP) studies have been one of the focal points of research worldwide, especially among development economists. While studies in the field of growth have long existed, there is always a quest to identify new ways to boost economic growth [1]. Many theories have been developed and continue to be developed to better understand economic growth drivers and their interrelations. Many variables have been researched to ascertain their possible explanation to economic growth through the many attempts to unearth economic growth drivers. Some of these readily essential factors in development economics literature include trade openness (TROP) [2, 3], Carbon dioxide emissions (CO_2_) [4, 5], energy consumption (ENR) [6, 7].

Two significant capital sources for developing countries especially Africa, to enhance economic growth are foreign Aid (FIAD) and foreign direct investment (FDI) [8]. Mainly FIAD flows to developing countries from developed countries in the form of official development assistance (ODA) to step up economic growth [8]. Thus, FIAD supplements other financing sources and creates the necessary international and domestic conditions to attract and facilitate the FDI inflows, which developing countries are unable to attract directly [9]. This Aid’s broad objective is to stimulate economic growth by providing educational infrastructure, stabilizing economies that have been afflicted by economic shocks, and financing health [10]. It should be stated that this could be achieved based on a well-developed market, a sound system of governance, and a trade-friendly policy [9]. Thus, FIAD increases capital’s marginal productivity and establishes conditions conducive to private investment and FDI by providing access to capital and technical skills [11]. These capitals and skills provided to the capital-scarce emerging economies cause dynamism for higher sustainable economic growth. [12] posit that FIAD helps develop complement social overhead capital (SOC) as dams for electricity generation and building roads. Thus, FIAD plays a pivotal role in economic growth, however, the attainment of sustainable economic development requires maximum energy consumption [5].

Energy consumption is considered an essential resource in supporting economic growth and sustainable development [13]. It is generally believed that energy paly an indispensable role in the process of economic and social development and enhanced the quality of life in develop and developing countries of which African economies are not exceptional [14]. Primary energy demand in Africa stood at over 830 million metric tons of oil equivalent (Mtoe) in 2018, and the demand was set to increase in 2019 [15]. Therefore, the role of energy in African economic development is without controversy, thus an insufficient supply of energy will affect all aspects of economic and social development [16]. Though energy consumption contributes significantly to economic growth, it is also viewed as a major underlying cause of greenhouse emissions and global warming [17]. Though the contribution of Africa to the global emissions is very small compared to China, Europe, and OECD countries, CO_2_ emissions rose from 669.7 million tons to 1058.3 million tons in 2011in Africa [18]. This is was vital to include ENR and CO_2_ emissions in this study.

Another determinant of economic growth is trade openness (TROP). Trade openness is said to be an integral component of economic development because economic activities demand energy consumption [19]. The influence on economic growth by trade openness has been determined by the nation’s magnitude of energy consumption [5]. Trade openness allows emerging economies to import from developed economies advanced technologies. These advanced technologies mostly emerge in developing countries through FIAD and FDI in official development assistance to increase economic growth. Thus, through trade, FDI and FIAD have significant impact on economic growth. This it inclusion in this study was vital. Lastly, considering the government’s assistance to the private sector to revive the industrial sector or increase productivity, it was necessary to consider domestic financial assistance (DFA) as a key factor of economic growth. [20] stated that DFA to the private sector is purposely aimed to nurture higher economic growth, likewise did [21] emphasized that FIAD increases government finance capacity, but it should be mentioned that FIAD inflows do not supplement domestic assistance. This is because there is no assurance that countries will use FIAD solely to fund domestic investment for industrialization [22]. Hence, DFA can be seen as a nation pursuing economic growth through higher domestic investments.

Based on the highlights above, the current study explores the key factors of economic growth in Africa, since countries in Africa are developing economies. This study is complementary to existing knowledge by accounting for other covariates like; Economic Growth, foreign aid, foreign direct investment, Energy Consumption, Trade openness, Domestic Financial Assistance, and CO_2_ emission in Africa. Therefore, this study attempt to bridge the identified gap in Africa as some previous documented study failed to do in two ways; (a) In terms of scope, this study, to the best of the authors’ knowledge is the first to examine the employed variables in these sub-panels; Arab Maghreb Union & East African Community, Community of Sahel-Saharan States, Common Market for Eastern and Southern Africa, Sothern African Development Community, Economic Community of West African States. Also, the study discusses the problems and opportunities for energy use sources, sustainable growth, and environmental degradation in Africa. (b) With regards to the methodological front, it is known fact cross-sectional dependence and slope homogeneity are well-known problems in panel econometrics, which previous studies have failed to solve. Thus, this study circumvents these problems in its econometric modeling setting. The study employs recent panel estimators to ensure reliable and consistent results which are worthwhile for decision making in concerned countries in Africa.

The remaining sections are as follows: the selected countries and variables in section 2, followed by section 3, theoretical model and specification and methodology. Empirical results in section 4, discussion in section 5, then finally section 6 with the conclusion and policy implication.

## 2. Literature review

This section is dedicated to analyzing the results of some studies using panel data modeling techniques on CO_2_ emissions, FDI, and the economic growth nexus. It should also be noted that, compared to modeling techniques based on time series data, panel data modeling techniques are relatively recent. In this context, as they are closest to our research, we focus on reviewing studies on panel data models and therefore provide at least some insights into the relationship between economic growth and its determinants.

[23] employed FDI and financial development as determinants of economic growth in Sudan using annual data from 1970 to 2014. By employing cointegration test and the fully modified ordinary least squares and dynamic ordinary least squares techniques, they revealed that financial development is more beneficial to economic growth than FDI. However, FDI leads to better economic performance through financial development. [24] Studied the relationship between economic growth, foreigh direct investment, environmental quality and financial development for the Middle East countries over the period 1980–2014. They employed the Cobb-Douglas production function and the simultaneous equation panel data model together with the GMM estimator. Their empirical findings revealed that FDI is a good determinant of economic growth however it decreases the quality of the environment. [25] examined linkages among FDI and economic growth in Central and Eastern Europe using 11 countries for the period of 1997–2014. Findings from panel data analysis suggest that the relative size of economic growth indicators affect FDI of these 11 countries. From their results, FDI has an impact on economic growth, and this effect is strengthened by financial market development. [9] determined the relationship between economic growth, FDI, and foreign aid in South Asia and South-East Asia for the period 1980–2016 by employing the GMM estimator. Their results indicated that foreign aid has a negative effect on economic growth, FDI positively influences growth. Their study again stated that governmental financial assistance to private sector is another important determinant of economic growth. [26] investigated the causal relationship between economic growth, urbanization and energy consumption in New Emerging Market countires from 1971 to 2014 by employing the Dimitrescu-Hurlin panel granger causality test. Their empirical findings revealed that a bidirectional cusation effect exist between urbanization and economic growth. [27] explored the relationship between urbanization, trade flow and economic growth in Nigeria from 1980 to 2016 by applying the ARDL bounds test and the the Bayer and Hanck cointegration test. Their empirical evidence indicated that urbanization is a major determinant of economic growth in Nigeria.

[28] explored the interrelationship between economic growth, energy consumption and environmental degradation in 35 OECD countries over the period 2000–2014 by using the generalized method of moments and the panel vector autoregressive regression. Their findings indicate that energy consumption is a major determinant of economic growth. [29] investigated the causal relationship between energy consumption and economic growth using 53 countries over a period 1990–2014 by employing the granger causality. The results obtain during the study revealed that a bidirectional causation exist between energy consumption and economic growth. [30] examined the interrelationship between energy consumption and economic growth in top ten energy consuming countries by employing the quantile-on-quantile eatimation panel approach. The results reavealed a positive association between economic growth and energy consumption. Their results again pointed that a weak effect of economic growth on energy consumption was felt in countries like USA and Canada. [31] explored the relationship between trade openesses and economic growth in China over the period 1994–2018 by employing the ARDL model. Evidence from their results indicated that a long-term stable cointegration relationship between trade openness and economic growth. Again, they revealed a positive effect of trade openness on economic growth. Finally, the result indicated an “N-type” relation between trade openness and economic growth. [32] explored the impact of trade openness on economic growth in five emerging market economies using a panel data from 1993 to 2016 by employing the Dimitrescu-Hurlin granger causality test. The empirical results confirm the long‐run association between trade openness and economic growth. The panel causality tests indicate the presence of a bidirectional causality between economic growth and trade openness. [33] explored the relationship between foreign aid, FDI and economic growth in over the period 1976–2015 by using the GMM estimator. An insignificant effect of foreign aid on economic growth was revealed. However, it was indicated that FDI has a significant positive effect on economic growth. Table 1 provides a summary of recent study on the determinants of economic growth in different locations.

**Table 1:** Recent empirical studies on the relationship among the variables.

Authors	Research Area	Duration	Methodology	Inference
[61]	Nigeria	1982–2016	VECM	Foreign aid has a significant effect on GDP.
[8]	Cambodia	1980–2014	ARDL	FIAD has a positive impact on growth for the short run.
[62]	74 developing countries	1980–2016	2SLS estimation	The marginal effect of foreign aid improves GDP.
[9]	Asian countries	1980–2016	System GMM	Foreign Aid positively influences growth.
[63]	25 developing countries	1984–2008	PSTR	A positive impact of aid flows on GDP was seen.
[64]	82 developing countries	1981–2013	VECM	A one-way causal effect from GDP to FIAD was seen.
[11]	45 SSA countries	1993–2017	FMOLS and PDOLS	A one-way causal effect from FIAD to GDP was seen.
[35]	11 European countries	2003–2016	VECM	Two-way causal effect between FDI and GDP
[65]	Fifteen Asian countries.	1990–2013	ARDL	A one-way causal effect from FDI to GDP was seen.
[34]	25 Sub-Saharan countries	1980–2014	GMM	FDI induce a positive effect on GDP
[66]	31 Chinese provinces	2000–2015	VECM	Unilateral causality from GDP and FDI
[20]	Spain	1984–2010	2SLS estimation	FDI stimulate economic growth in Spain
[67]	Europe	2012–2014	Block exogeneity	FDI is a critical factor in accelerating GDP
[68]	169 countries	1988–2014	GMM	Openness to trade significantly impact growth
[69]	Turkey	1960–2013	VECM	Unilateral causality from trade openness to GDP
[70]	34 SSA countries	1996–2016	SEM	Trade openness has a positive impact on GDP
[71]	15 Asian countries	1990–2017	VECM	Trade has a positive impact on GDP
[72]	BRICS	1966–2015	Granger causality	Two-way causal effect between TROP and GDP
[73]	China	1994–2018	ARDL	The effect of trade openness on GDP positive
[74]	33 European countries	1996–2017	Granger causality	Energy has a significant impact on GDP
[75]	5 OPEC countries	1990–2014	FMOLS/ DOLS	A positive effect of energy was felt on GDP
[76]	Pakistan	1975–2016	Robust least square	Energy has a significant impact on GDP
[6]	BRI	1995–2015	D-H causality test	A unidirectional casusation effect of ENR was felt on GDP
[52]	BRI	1991–2018	AMG and CCEMG	Unidirectional causal effect from GDP to CO_2_
[51]	186 countries	1980–2015	Granger causality	A bidirectional causal effect between GDP and ENR
[1]	China	1996–2015	VAR approaach	Energy consumption can boost economic growth
[77]	Italy	1960–2011	VECM	Unidirectional causal effect from GDP to CO_2_

**Note:** CO_2_ = CO_2_ emissions, GDP = Economic growth, FIAD = Foreign aid, FDI = Foreign direct investment, TROP = Trade openness, ARDL = autoregressive distributed lag, GMM = generalized method of moments, SEM = Structural Equation Model, AMG: Augmented mean group, CCEMG = Common correlated effects mean group, PSTR = Panel Smooth Transition Regression Model, SEM = Structural equation modelling, SEASA = South easth Asia and Southern Asia, GCC = Gulf Cooperation Council, BRI = Belt and Road Initiative, E.C = Electricity consuming countries, BRICS = Brazil, Russia, India, China and South Africa, OECD = Organization for Economic Co-operation and Development.

Overall, our literature review suggests that the empirical results of the previous are inconclusive. A potential reason is that previous did not consider the linkage between foreign aid, foreign direct investment, economic growth, trade openness, domestic financial assistance, energy consumption, and CO_2_ emissions jointly for the ECOWAS community. Thus in this study, we address this void by applying recent panel estimators to a panel data set in African countries, taking into consideration the six major trading blocs.

## 3. Data, model specification, and methodology

### 3.1. Data and variable selection

Africa has six major trading blocs: East African Community, Arab Maghreb Union, Common Market for Eastern and Southern Africa, Sothern African Development Community, Economic Community of West African States, and Community of Sahel-Saharan States, with their members presented in Table 2. East African Community and Arab Maghreb Union were put together due to their same size. The time frame 1990 to 2018 was selected due to the availability of data. Table 3 shows the series selected, definition, abbreviation, and sources.

**Table 2:** List of selected countries in various trading blocs.

African Regional Economic Blocks	Abbreviation	Selected countries
Arab Maghreb Union & East African Community	**AMU-EAC**	Algeria, Burundi, Kenya, Tanzania, Uganda, Rwanda, Mauritania, Morocco, Tunisia
Community of Sahel-Saharan States	**CEN-SAD**	Benin, Burkina Faso, Cote d’Ivoire, Egypt, Gambia, Ghana, Mali, Mauritania, Morocco, Nigeria, Senegal, Sierra Leone, Sudan, Togo, Tunisia,
Common Market for Eastern and Southern Africa	**COMESA**	Burundi, Egypt, Kenya, Malawi, Madagascar, Mauritius, Rwanda, Sudan, Seychelles, Eswatini, Uganda, Zimbabwe, Zambia,
Sothern African Development Community	**SADC**	Angola, Botswana, Lesotho, Madagascar, Malawi, Mauritius, Mozambique, Seychelles, Eswatini, South Africa, Zimbabwe, Zambia
Economic Community of West African States	**ECOWAS**	Benin, Burkina Faso, Cote d’Ivoire, Gambia, Ghana, Mali, Nigeria, Senegal, Sierra Leone, Togo

**Table 3:** Variables description and data source.

Variable	Definition	Abbreviation	Period	Source
Economic Growth	GDP per capita (constant 2010 US$)	GDP	1990–2018	WDI
Domestic Financial Assistance	domestic credit provided by government to private sector	DFA	1990–2018	WDI
Energy Consumption	Energy consumption per capital (kg of oil equivalent)	ENR	1990–2018	WDI
Foreign Direct Investment	Foreign direct investment inflows	FDI	1990–2018	WDI
Foreign Aid	Net official Aid refers to countries and territories	FIAD	1990–2018	WDI
Carbon Dioxide Emissions	CO_2_ emissions (metric tons per capita)	CO_2_	1990–2018	WDI
Trade openness	Sum of imports and exports as a percentage of GDP.	TROP	1990–2018	WDI

### 3.2. Model specification

In this study, GDP was the dependent variable, while FIAD, FDI, ENR, DFA, CO_2_, and TROP were the explanatory variables used as determinants of GDP in Africa’s trading blocs. Thus, following the work of [34–36] the function for the study was modeled as below;

$$\text{GDP}=\text{F}(\text{FIAD},\text{FDI},\text{ENR},\text{DFA},{\text{CO}}_{2},\text{TROP})$$ (1)

Therefore, to resolve the issue of the lack of homoscedasticity, the transformed multivariate GDP model was written as:

$${LnGDP}_{it}={\beta_{0}+\beta_{1}{LnFIAD}_{it}+{\beta_{2}{LnENR}_{it}+\beta_{3}{LnCO}_{2it}+\beta_{4}\;{LnTROP}_{it}+\beta }_{5}{LnFDI}_{it}+\beta }_{6}{LnDFA}_{it}+\varepsilon_{it}$$ (2)

Where i stands for each selected countries in the study (1, 2… N), error term giving by ε_it_, t for the period of study and β_0_ is the slope coefficient. β_1_, β_2_, β_3_, β_4_, β_5_, and β_6_ are the coefficients for FIAD, ENR, CO_2_, TROP, FDI, and DFA, respectively.

### 3.3. Methodology

#### 3.3.1. Cross-sectional dependency

Because of the African countries’ interconnectedness regarding activities such as trade, the data from these countries will likely indicate a strong interdependency. Therefore, ignoring the cross-section dependency in the analysis panel data will lead to error estimation. Thus, cross-sectional dependency tests such as the CD test from [37], the bias correction LM test from [38], the scale LM test from [39], and the LM test from [40] were employed. The standard model for panel data can be stated as;

$$y_{i,t}=\alpha_{i}+\beta_{i,t}X_{i,t}+\mu_{i,t}$$ (3)

Where i = 1,2, … …N, β_it_ being a K × 1 parameter vector to be calculated, x_it_ is a K × 1 independent variable, t = 1,2, … … T, μ_it_ is it time-invariant to assume independent and identical distributions and α_1_ is the individual redundant parameter. The null hypothesis of the non-existence of cross-sectional correlation against the alternate hypothesis of the existence of cross-sectional correlation can be written as below:

$${\text{H}}_{0}=\rho_{\text{ij}}=\rho_{\text{ji}}=\text{cor}\left(\mu_{\text{it}},\mu_{\text{jt}}\right)=0\;\text{for}\;\text{i}\neq \text{j}$$ (4a)

$${\text{H}}_{1}=\rho_{\text{ij}}=\rho_{\text{ji}}=\text{cor}\left(\mu_{\text{it}},\mu_{\text{jt}}\right)\neq 0\;\text{for}\;\text{some}\;\text{i}\neq \text{j}$$ (4b)

Where in the model and calculated as below, the product correlation coefficient incurred from the error terms is ρ_ij_ or ρ_ji_. Again, with N, the number of potential pairings (μ_it_, μ_jt_) increases.


$$\rho_{ij}=\rho_{ji}=\frac{\sum_{t=1}^{T}\mu_{it}\mu_{jt}}{{{\left(\sum_{t=1}^{T}{\mu_{it}}^{2}\right)}^{1/2}(\sum_{t=1}^{T}{\mu_{jt}}^{2})}^{1/2}}$$ (5)

The [40] LM test may be employed to test the interdependence of heterogeneous panels if T goes to infinity and n is constant. The test is determined using the expression;

$${LM}_{BP}=T\sum_{i=1}^{n-1}\sum_{j=i+1}^{n}{\overset{ˇ}{\rho }}_{ij}^{2}$$ (6)

The [40] LM test is distributed asymptotically with *χ*^2^ of degrees of Freedom *n*(*n* − 1)/2. However, as n goes to infinity, it is not feasible to apply this test. The scaled version of test LM_BP_ proposed [39] the test was employed and it is expressed as;

$${CD}_{Lm}=\sqrt{\frac{1}{n(n-1)}}\sum_{i=1}^{n-1}\sum_{j=i+1}^{n}{(T\overset{ˇ}{\rho }}_{ij}^{2}-1)$$ (7)

The CD_Lm_ as shown by [39] is the asymptotic distribution as N(0,1) as N → ∞ with T→ ∞. The bias correction LM test statistics was proposed by [38] and given as below;

$${LM}_{BC}={LM}_{P}-\frac{n}{2\left(T-1\right)}=\sqrt{\frac{1}{n(n-1)}}\sum_{i=1}^{n-1}\sum_{j=i+1}^{n}\left(T\rho_{ij}^{2}-1\right)-\frac{n}{2(T-1)}$$ (8)

The limiting distribution of the bias correction LM test is usually distributed with the null. This hypothesis is stated as follows:

$$H_{0}=\rho_{ij}=0\;for\;i\neq j$$ (9)

*ρ*_*ij*_ is the correlation coefficient of the error terms where $\rho_{ij}=\frac{\sigma_{ij}}{\sqrt{{\sigma_{i}}^{2}{\sigma_{j}}^{2}}}$. With the alternative hypothesis of a non-zero correlation coefficient *H*_1_ = *ρ*_*ij*_ ≠ 0, for some *i* ≠ *j*. Eventually, with the null hypothesis that the error term has a weak cross-sectional dependence, [37] proposed the CD test statistic, which can be expressed as;

$$CD=\sqrt{\frac{2T}{N\left(N-1\right)}}\left(\sum_{i=1}^{N-1}\sum_{j=i+1}^{N}{\overset{ˇ}{\rho }}_{ij}\right)$$ (10)

$${\hat{\rho }}_{ij}={\hat{\rho }}_{ji}=\frac{\sum_{t=1}^{T}{\hat{u}}_{it}{\hat{u}}_{jt}}{{\left({\sum_{t=1}^{T}\hat{u}}_{it}^{2}\right)}^{1/2}{\left({\sum_{t=1}^{T}\hat{u}}_{jt}^{2}\right)}^{1/2}}$$ (11)

With ${\hat{\rho }}_{ij}$ being the coefficient of correlation. If the error term of unit i in period t is u_i_, then the hypothesis of the test can be expressed as;

$$H_{0}=E\left(u_{it}u_{jt}\right)=0,{⩝}_{t}\;and\;i\neq j$$ (12)

For the unbalanced panel, the formula for calculating the correlation coefficient is;

$${\hat{\rho }}_{ij}={\hat{\rho }}_{ji}=\frac{\sum_{t\epsilon T_{i}T_{j}}\left({\hat{u}}_{it}-\begin{array}{c} -\\ {\hat{u}}_{i} \end{array}\right)\left({\hat{u}}_{jt}-\begin{array}{c} -\\ {\hat{u}}_{j} \end{array}\right)}{{\left[\sum_{t\epsilon T_{i}{\cap T}_{j}}{\left({\hat{u}}_{it}-\begin{array}{c} -\\ {\hat{u}}_{i} \end{array}\right)}^{2}\right]}^{1/2}{\left[\sum_{t\epsilon T_{i}\cap T_{j}}{\left({\hat{u}}_{jt}-\begin{array}{c} -\\ {\hat{u}}_{j} \end{array}\right)}^{2}\right]}^{1/2}}$$ (13)

Where;

$\begin{array}{c} -\\ {\hat{u}}_{j} \end{array}=\frac{\sum_{t\epsilon T_{i}{\cap T}_{j}}{\hat{u}}_{it}}{T_{ij}},T_{ij}=(T_{i}=T_{j})$ hence the CD test statistic turns;

$$CD=\sqrt{\frac{2}{N\left(N-1\right)}}\left(\sum_{i=1}^{N-1}\sum_{j=i+1}^{N}\sqrt{T_{ij}}{\hat{\rho }}_{ij}\right)$$ (14)

The CD test statistic is distributed asymptotically under the null as it becomes normal as *CD* ~ N(0,1).

#### 3.3.2. Slope heterogeneity test

The [41] test was employed to investigate whether there is heterogeneity in slope coefficients because ignoring the presence of slope heterogeneity might not be conducive to regression analysis. This test statistic can be computed via the relation;

$$\tilde{S}=\sum_{i-1}^{N}{\left({\tilde{\beta }}_{i}-{\tilde{\beta }}_{WFE}\right)}^{\prime }\frac{x_{i}^{\prime }M_{\tau }x_{i}}{{\tilde{\sigma }}_{i}^{2}}\left({\tilde{\beta }}_{i}-{\tilde{\beta }}_{WFE}\right)$$ (15)

$$\tilde{\Delta }=\sqrt{N}\left(\frac{N^{=1}\tilde{S}-k}{\sqrt{2k}}\right)$$ (16)

where the test statistics are $\tilde{\Delta }$ and $\tilde{S}$, the pooled OLS coefficient being $\tilde{\beta },{\tilde{\sigma }}_{i}^{2}$ is the estimate of $\sigma_{i}^{2}$, the weighted fixed effect pooled estimator being ${\tilde{\beta }}_{WFE}$, *M*_*τ*_ being the identity matrix, *x*_*i*_ being the matrix carrying input series in difference from the mean and *k* is the number of predictors.

The $\tilde{\Delta }$ test’s biased-adjusted version is expressed as;

$${\tilde{\Delta }}_{adj}=\sqrt{N}\left(\frac{N^{-1}SE({\tilde{z}}_{iT})}{\sqrt{Var({\tilde{z}}_{iT})}}\right)$$ (17)

Where;

$$E\left({\tilde{z}}_{iT}\right)=k,Var\left({\tilde{z}}_{iT}\right)=\frac{2k(T-k-1)}{T+1}$$ (18)

#### 3.3.3. Panel unit root test

In view of the fact that CSD was characterized in the panel data, it is important to consider unit roots test considered by CSD to obtain accurate estimates. In response to this, the [42] cross-sectionally dependent augmented dickey-fuller (CADF) which is a second-generation panel root test was adopted was. For this test, the regression is given as:

$$\Delta y_{it}=\alpha_{i}+\beta_{i}y_{it-1}+\theta_{i}{\overset{-}{y}}_{t-1}+\sum_{j=1}^{\rho }\gamma_{ij}\Delta y_{it-1}+\sum_{j=0}^{\rho }\delta_{ij}\Delta {\overset{-}{y}}_{t-j}+d_{it}+\varepsilon_{it}$$ (19)

Where ${\overset{-}{y}}_{t}=\frac{1}{N}\sum_{i=1}^{N}y_{it}$ and it is included in the equation as a substitute for the unnoticed common factors effects. *β*_*i*_, *θ*_*i*_, *γ*_*ij*_, *and δ*_*ij*_ respectively represent an individual-specific linear trend, common time effect and individual-specific effect. *α*_*i*_ is a time-invariant individual nuisance parameter. After running the CADF statistic, the CIPS, which similar to the IPS statistic of [43], it is computed as;

$$CIPS\left(N,T\right)=\frac{1}{N}\sum_{i=1}^{N}t_{i}(N,T)$$ (20)

Where *t*_*i*_(*N*, *T*) is the value of *β*_*i*_ in Eq 18. ${\overset{-}{y}}_{t}$ inclusion in the unit root equation makes the test statistic inconsistent with the ADF statistics, so Pesaran provides the critical values.

#### 3.3.4. Westerlund-Edgerton Co-integration test

To estimate the long-run effects of the parameters, the Westerlund-Edgerton bootstrap panel co-integration test was used. They suggested a two-panel co-integration test, taking into account the structural break in the co-integration slope and intercept for the null hypothesis of no co-integration. This test offers good results and applies to all cases where there is or no CSD. To assess the null hypothesis of no co-integration, [44] suggested four residual test methods. Two of the tests being panel statistics, and the other two being group statistics, which are normally distributed. Essentially, this test assesses the presence of co-integration by determining whether error correction occurs in a whole panel group panel and a particular group. This model was built on:

$$y_{it}=\delta_{0i}+\delta_{1it}+n_{i}D_{it}+{x^{\prime }}_{it}\beta_{i}+{\left(D_{it}x_{it}\right)}^{\prime }\gamma_{i}+z_{it}$$ (21)

Where D_it_ represent the break dummy variables,$T_{i}^{b}$ corresponds to the break date for individual i such that $T_{i}^{b}=\theta_{i}^{b}$ with $\theta_{i}^{b}\in (\Psi ,1-\Psi )$, x_it_ = x_i,t−1_ + v_it_ is k-dimensional vector being I(1), Ψ ∈ (0,1), δ_oi_ and n_i_ are unknown coefficient vectors, D_it_ = 1 if $t>T_{i}^{b}$ and zero otherwise, t = 1 … … T, i = 1 … … N, and z_it_ is the residual term. z_it_ is then develop as;

$$\vartheta_{i}\left(L\right)\Delta e_{it}=\vartheta_{i}e_{i.t-1}+\varepsilon_{it}$$ (22)

With $\vartheta_{i}\left(L\right)=1-\sum_{j=1}^{pi}\vartheta_{ij}L^{j}$ being a scalar lag polynomial and ε_it_ is the error process.

H_0_ = ϑ_i_ = 0 No existence of cointegration for ∀_i_
H_1_ = ϑ_i_ < 0 Existence of cointegration for ∀_i_

The alternate hypothesis during the testing implies that the transition to equilibrium is uniform among different populations. Rejecting the null hypothesis thus suggest the presence of cointegration in the various groups.

H_0_ = ϑ_i_ = 0 No existence of cointegration for ∀_i_
H_1_ = ϑ_i_ < 0 Existence of cointegration for ∀_i_

The alternate hypothesis means that there is a heterogeneous adjustment for equilibrium between different groups. The rejection of the null hypothesis indicates that there is an indication of cointegration among group members. Where ${\hat{a}}_{\hat{a}}$ is the standard error of ${\hat{a}}_{i}$, such that both the statistics diverged to negative infinity, indicating the test decisions were done based on the left tail of the standard normal distribution such that;

$$\hat{a}={\left(\sum_{i=1}^{N}\sum_{t=2}^{T}{\tilde{y}}_{i,t-1}\right)}^{-1}\sum_{i=1}^{N}\sum_{t=2}^{T}\frac{1}{{\hat{a}}_{i}}{\tilde{y}}_{i,t-1}\Delta {\tilde{y}}_{it}$$ (23)

a^a^=1N∑i=1Na^ia^i(1)2-1∑i=1N∑t=2Ty~2i,t-1-12 (24)

#### 3.3.5. Parameter estimation

This study used the Dynamic Seemingly Unrelated Regression (DSUR) estimator proposed by [45] to determine the independent variables’ effects on the dependent variable. For balanced panels where the number of co-integrating regression equations N is considerably smaller compared to the number of time-series observations T, the DSUR approach is feasible. The DSUR achieves efficiency improvements over non-system methods such as dynamic ordinary least square (DOLS) when heterogeneous sets of regressors join the regressions and when equilibrium errors are associated through cointegration regressions. Another advantage of the DSUR is that it can be employed when the panel is heterogeneous or homogeneous. The model is composed as;

$$y_{it}=\beta_{i}x_{it}+u_{it}^{Ϯ}$$ (25)

$$u_{it}^{Ϯ}=\rho_{i}u_{it-1}^{Ϯ}+\sum_{j=1}^{n-1}\delta_{ij}\Delta x_{it-1}+\omega_{it}$$ (26)

$$\Delta x_{it}=\emptyset_{i}\Delta x_{it-1}+m_{it}$$ (27)

$$\omega_{it}=\lambda_{i}\theta_{t}{+\zeta }_{it}$$ (28)

Where *x*_*it*_ is the *k* × 1 dimensional vector for the exploratory variables, the cross-sectional endogeneity, and cross-sectional dependency are inflected by varying λ_i_ and *ρ*_*i*_. *x*_*it*−1_ was included to control the problem endogeneity. $u_{it}^{Ϯ}=(u_{1t}^{Ϯ},\ldots \ldots u_{Nt}^{Ϯ})$, $\omega_{it}={\left(u_{it}^{Ϯ}\right)}^{\prime }$ is a dimensional vector *N*(*K* = 1) with a moving average representation of the orthonormal Wold.

#### 3.3.6 Causality test

Eventually, because the DSUR estimator could not prove the analyzed sequence’s causation, the [46] causality test was employed. The estimator is employed because of its robustness in slope heterogeneity of slope and cross-section dependence. The Dumitrescu-Hurlin test is modeled by the expression below:

$$y_{i,t}=\alpha_{i}+\sum_{k=1}^{K}\gamma_{i}^{\left(k\right)}y_{i,t-k}+\sum_{k=1}^{K}\beta_{i}^{\left(k\right)}x_{i,t-k}+\varepsilon_{i,t}$$ (29)

Where the lag order is denoted by *K* and presumed to be the same for all cross-sectional units $\gamma_{i}^{\left(k\right)}$ and $\beta_{i}^{\left(k\right)}$ denote lag parameters and slope that distinguish across groups. The individual effects of countries donated by *α*_*i*_, $\beta_{i}={\left(\beta_{i}^{\left(1\right)},\ldots ,\beta_{i}^{\left(k\right)}\right)}^{\prime }.$

## 4. Empirical results

### 4.1. Exploratory data analysis

#### 4.1.1. Descriptive statistics

The transformation of the data was done using natural logarithm so to explain the coefficients of the variables as elasticities. As unveil in Table 4, AMU-EAC has GDP (SD = 0.880, M = 6.950), DFA (SD = 0.832, M = 2.813), ENR (SD = 0.750, M = 6.250), FDI (SD = 3.262, M = 18.274), FIAD (SD = 0.819, M = 20.291), CO2 (SD = 1.926, M = 8.512), TROP (SD = 1.203, M = 15.700). CEN-SAD has GDP (SD = 0.616, M = 7.025), DFA (SD = 0.843, M = 2.672), ENR (SD = 0.734, M = 5.991), FDI (SD = 2.317, M = 18.534), FIAD (SD = 0.958, M = 20.124), CO2 (SD = 1.786, M = 8.515), TROP (SD = 1.391, M = 15.518). COMESA has GDP (SD = 1.123, M = 6.986), DFA (SD = 0.772, M = 2.669), ENR (SD = 0.996, M = 6.246), FDI (SD = 2.449, M = 17.889), FIAD (SD = 1.511, M = 19.903), CO_2_ (SD = 1.646, M = 7.897), TROP (SD = 1.528, M = 15.188). SADC has GDP (SD = 1.159, M = 7.553), DFA (SD = 0.921, M = 2.875), ENR (SD = 1.044, M = 6.628), FDI (SD = 1.818, M = 18.801), FIAD (SD = 1.453, M = 19.322), CO2 (SD = 1.756, M = 8.193), TROP (SD = 1.505, M = 14.970). ECOWAS has GDP (SD = 0.490, M = 6.737), DFA (SD = 0.472, M = 2.447), ENR (SD = 0.748, M = 5.762), FDI (SD = 2.133, M = 18.300), FIAD (SD = 0.957, M = 19.982), CO2 (SD = 1.530, M = 7.879), TROP (SD = 1.412, M = 15.3512).

**Table 4:** Descriptive statistics.

Panels	Variable	Mean	Std.Dev	Skewness	Kurtosis	JB test
**AMU-EAC**	GDP	6.950	0.880	-0.047	2.039	10.134^**a**^
	DFA	2.813	0.832	0.137	2.326	5.745^**c**^
	ENR	6.250	0.750	0.726	5.054	68.851^**a**^
	FDI	18.274	3.262	-1.434	4.775	123.825^**a**^
	FIAD	20.291	0.819	-0.137	2.407	4.642^**c**^
	CO_2_	8.512	1.926	0.027	1.859	14.188^**a**^
	TROP	15.700	1.203	-1.039	4.952	88.408^**a**^
**CEN-SAD**	GDP	7.025	0.616	0.010	2.394	6.654^**b**^
	DFA	2.672	0.843	-0.058	2.758	5.304^**c**^
	ENR	5.991	0.734	-1.289	5.453	229.622^**a**^
	FDI	18.534	2.317	-0.552	2.969	22.115^**a**^
	FIAD	20.124	0.958	-0.220	3.260	4.768^**c**^
	CO_2_	8.515	1.786	0.329	2.219	18.935^**a**^
	TROP	15.518	1.391	-0.608	3.748	36.961^**a**^
**COMESA**	GDP	6.986	1.123	0.523	2.424	24.121^**a**^
	DFA	2.669	0.772	0.027	3.402	21.788^**a**^
	ENR	6.246	0.996	1.597	6.058	330.979^**a**^
	FDI	17.889	2.449	-1.118	4.870	143.826^**a**^
	FIAD	19.903	1.5112	-1.008	3.506	73.165^**a**^
	CO_2_	7.897	1.646	0.739	3.427	40.105^**a**^
	TROP	15.188	1.528	-0.656	3.012	29.158^**a**^
**SADC**	GDP	7.553	1.159	-0.095	1.680	25.753^**a**^
	DFA	2.875	0.921	0.642	3.103	24.103^**a**^
	ENR	6.628	1.044	1.005	3.914	70.775^**a**^
	FDI	18.801	1.818	0.261	2.456	8.232^**b**^
	FIAD	19.322	1.453	-0.606	2.609	23.528^**a**^
	CO_2_	8.193	1.756	1.429	4.872	169.335^**a**^
	TROP	14.970	1.505	-0.562	2.873	18.573^**a**^
**ECOWAS**	GDP	6.737	0.490	-0.033	2.427	4.809^**c**^
	DFA	2.447	0.472	-0.591	3.227	17.546^**a**^
	ENR	5.762	0.748	-1.363	4.635	122.124^**a**^
	FDI	18.300	2.133	-0.179	2.889	4.701^**c**^
	FIAD	19.982	0.957	-0.392	3.064	7.488^**b**^
	CO_2_	7.879	1.530	0.637	3.028	19.668^**a**^
	TROP	15.3512	1.412	-0.259	3.208	31.783^**a**^

To access the normality of the employed variables, kurtosis and skewness was used. A variable must assume a value of 3 and 0 for kurtosis and skewness, respectively, to be considered as normally distributed. In AMU-EAC, GDP, FDI, FIAD, and TROP are negatively skewed, with DFA, ENR, and CO_2_ been positively skewed. In contrast, ENR, FDI, and TROP are fatter-tailed Kurtosis (K > 3), as GDP, DFA, FIAD, and CO_2_ unveiled a thinner tailed distribution with kurtosis (K < 3). In CEN-SAD, GDP and CO_2_ are positively skewed, as DFA, ENR, FDI, FIAD, and TROP are skewed to the left. GDP, DFA, FDI, and CO_2_ are thinner tailed with kurtosis (K < 3), as ENR, FIAD, and TROP are fatter-tailed Kurtosis (K > 3). In COMESA, GDP, DFA, ENR, FIAD, and CO_2_ are positively skewed with FDI, FAID and TROP been positively skewed, whereas DFA, ENR, FDI, FIAD, CO_2_, and TROP been fatter tailed Kurtosis (K > 3), as GDP showed a thinner tailed distribution with kurtosis (K < 3). In SADC, GDP, FIAD, and TROP are negatively skewed, as DFA, ENR, FDI, and CO_2_ are skewed to the right. GDP, FDI, FAID, and TROP are thinner tailed with kurtosis (K < 3), as DFA, ENR, and CO_2_ are fatter-tailed Kurtosis (K > 3). In ECOWAS, CO_2_ is positively skewed, as GDP, DFA, ENR, FDI, FIAD, and TROP are skewed to the left. DFA, ENR, FIAD, CO_2_, and TROP are fatter tailed with kurtosis (K > 3), as GDP and FDI are thinner tailed Kurtosis (K < 3). Hence none of the employed variables were normally distributed. The rejection of the above variables’ normality distribution is supported strongly by the Jarque-Bera (JB) normality test. The JB test points out that all variables are not normally distributed when the rejection probability is less than 0.05.

#### 4.1.2. Correlation among the employed variables

**Fig 1:**
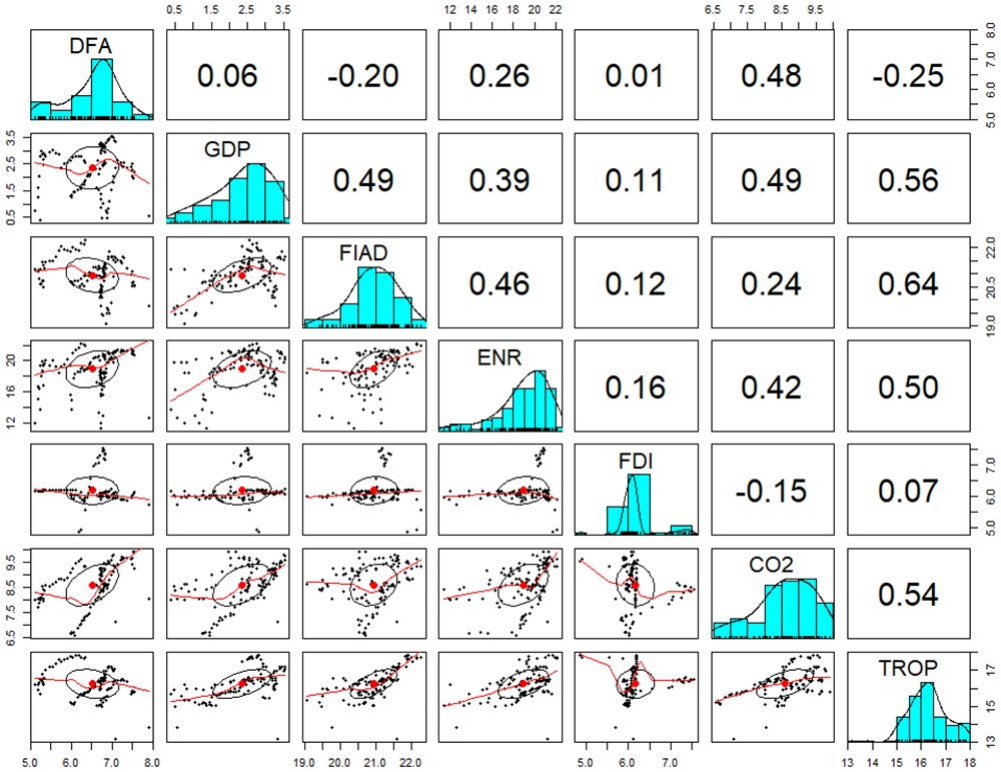
Correlation plot among the employed variables.

The relationships among the employed variables can be depicted in Fig 1. The scatter plot unveils the direction among the variables with GDP. FAID, CO_2_, and TROP showed a positive trend with GDP. FDI, DFA, and ENR showed no specific trend with GDP. From Fig 1, no strong correlation among the explanatory variables was indicated since the coefficient of correlation among the variables was less than 0.7. In calculating the correlation, the Pearson product difference correlation is given formula as;

$$r=\frac{\sum_{i=1}^{n}\left(x_{1i}-{\hat{x}}_{1})(x_{2i}-{\hat{x}}_{2}\right)}{\sqrt{\sum_{i=1}^{n}{\left(x_{1i}-{\hat{x}}_{1}\right)}^{2}}\sqrt{\sum_{i=1}^{n}{\left(x_{2i}-{\hat{x}}_{2}\right)}^{2}}}$$ (30)

### 4.2. Cross-sectional dependency results

The findings of the cross-section dependence test are shown in Table 5. The results from the various test indicated that the null hypothesis of cross-sectional independence is dismissed. The inference was there is enough evidence of cross-section dependence in the error terms among the panels. Hence econometric estimations that consider cross-sectional dependence was employed.

**Table 5:** Cross sectional dependence results.

	AMU-EAC		CENSAD		COMESA		SADC		ECOWAS	
	Value	Prob.	Value	Prob.	Value	Prob.	Value	Prob.	Value	Prob.
**Breusch-Pagan LM**	53.66^**b**^	0.029	165.4^**a**^	0.000	166.94^**a**^	0.000	88.89^**b**^	0.031	83.42^**a**^	0.004
**Bias-corrected scaled LM**	3.816^**a**^	0.000	8.088^**a**^	0.000	11.71^**a**^	0.000	3.266^**a**^	0.001	8.523^**a**^	0.000
**Pesaran scaled LM**	3.152^**a**^	0.000	3.677^**a**^	0.000	3.272^**a**^	0.001	2.023^**b**^	0.043	2.73^**a**^	0.006
**Pesaran CD**	26.251^**a**^	0.000	14.641^**a**^	0.000	21.524^**a**^	0.000	15.820^**a**^	0.003	8.027^**a**^	0.002
**Friedman**	31.238^**a**^	0.000	27.511^**a**^	0.016	31.135^**a**^	0.003	31.126^**a**^	0.001	50.728^**a**^	0.000

**Note:** 1%, 5%, 10% level of statistical significance is represented by “a, b, and c” respectively.

### 4.3. Homogeneity test results

Ignoring heterogeneity of slope coefficients may lead to imprecise estimates and skewed inferences [47]. Hence, the [41] test was utilized in making that accession. As revealed in Table 6, the alternative hypothesis of heterogeneity in the slope coefficient is accepted. On this basis, estimators which are robust to heterogeneous problems and cross-sectional reliance were employed.

**Table 6:** Pesaran and Yamagata homogeneity test results.

	AMU-EAC		CEN-SAD		COMESA		SADC		ECOWAS	
	Value	Prob.	Value	Prob.	Value	Prob.	Value	Prob.	Value	Prob.
**Delta tilde ($\tilde{\Delta }$)**	11.24^**c**^	0.021	6.104^**a**^	0.000	7.754^**a**^	0.000	5.278^**a**^	0.001	17.221^**b**^	0.012
**Adjusted delta tilde (${\tilde{\Delta }}_{adj}$)**	24.32^**a**^	0.000	31.78^**b**^	0.052	21.026^**a**^	0.001	32.055^**a**^	0.000	25.144^**a**^	0.000

**Note:** “**a, b** and **c”** imply significance at the 1%, 5%, and the 10% levels, respectively.

### 4.4. Panel unit root test results

Table 7 displays the results from the unit root test. The findings show that the null hypothesis of unit root is recognized irrespective of the time trend and levels. The seven variables were stable at the various significance levels 1%, 5%, and 10% after the first difference. Thus, in econometrics, it is deduced that the variables were of order 0, I (0), and then order 1, I (1). This then offers the possibility of further analysis of the long-term equilibrium relationship between the variables (GDP, DFA, ENR, FDI, FIAD, CO_2_, and TROP). As stated by [48], variables have to be stable to obtain efficient estimates.

**Table 7:** Panel Unit Root test results.

Variables	CIPS						CADF					
	Levels		Inf.	First difference		Inf.	Levels		Inf.	First difference		Inf.
	Constant	Constant &Trend		Const ant	Constant &Trend		Constant	Constant &Trend		Const ant	Constant &Trend	
**AMU-EAC**												
**GDP**	-1.200	-1.469	**I(0)**	-4.242^**a**^	-5.212^**a**^	**I(1)**	-1.605	-1.222	**I(0)**	-4.731^**a**^	-4.809^**a**^	**I(1)**
**DFA**	-1.697	-1.385	**I(0)**	-5.403^**a**^	-5.307^**a**^	**I(1)**	-1.302	-1.407	**I(0)**	-3.977^**a**^	-5.012^**a**^	**I(1)**
**ENR**	-1.205	-1.261	**I(0)**	-4.736^**a**^	-5.340^**a**^	**I(1)**	-1.622	-1.308	**I(0)**	-4.974^**a**^	-4.905^**a**^	**I(1)**
**FDI**	-1.112	-1.114	**I(0)**	-5.128^**a**^	-5.051^**a**^	**I(1)**	-1.342	-1.571	**I(0)**	-5.518^**a**^	-4.702^**a**^	**I(1)**
**FIAD**	-1.051	-1.154	**I(0)**	-4.830^**a**^	-5.245^**a**^	**I(1)**	-1.353	-1.400	**I(0)**	-3.955^**a**^	-5.004^**a**^	**I(1)**
**CO**_**2**_	-1.614	-1.329	**I(0)**	-5.540^**a**^	-4.502^**a**^	**I(1)**	-1.210	-1.328	**I(0)**	-4.017^**a**^	-4.571^**a**^	**I(1)**
**TROP**	-1.144	-1.510	**I(0)**	-4.301^**a**^	-4.713^**a**^	**I(1)**	-1.412	-1.308	**I(0)**	-4.129^**a**^	-4.620^**a**^	**I(1)**
**CEN-SAD**												
**GDP**	-1.103	-1.821	**I(0)**	-5.132^**a**^	-5.484^**a**^	**I(1)**	-1.316	-1.273	**I(0)**	-5.017^**a**^	-5.212^**a**^	**I(1)**
**DFA**	-1.093	-1.315	**I(0)**	-4.917^**a**^	-5.105^**a**^	**I(1)**	-1.135	-1.304	**I(0)**	-4.593^**a**^	-4.773^**a**^	**I(1)**
**ENR**	-1.212	-1.502	**I(0)**	-5.030^**a**^	-5.216^**a**^	**I(1)**	-1.291	-1.423	**I(0)**	-4.708^**a**^	-4.975^**a**^	**I(1)**
**FDI**	-1.341	-1.409	**I(0)**	-5.023^**a**^	-4.941^**a**^	**I(1)**	-1.233	-1.181	**I(0)**	-4.769^**a**^	-5.401^**a**^	**I(1)**
**FIAD**	-1.372	-1.407	**I(0)**	-5.113^**a**^	-5.321^**a**^	**I(1)**	-1.511	-1.031	**I(0)**	-5.303^**a**^	-4.750^**x**^	**I(1)**
**CO**_**2**_	-1.501	-1.704	**I(0)**	-4.708^**a**^	-4.798^**a**^	**I(1)**	-1.314	-1.327	**I(0)**	-5.217^**a**^	-4.925^**a**^	**I(1)**
**TROP**	-1.431	-1.624	**I(0)**	-4.921^**a**^	-5.091^**a**^	**I(1)**	-1.345	-1.217	**I(0)**	-5.175^**x**^	-5.109^**a**^	**I(1)**
**COMESA**												
**GDP**	-1.312	-1.213	**I(0)**	-5.209^**a**^	-5.024^**a**^	**I(1)**	-1.531	-1.325	**I(0)**	-5.011^**a**^	-4.259^**a**^	**I(1)**
**DFA**	-1.112	-1.144	**I(0)**	-4.931^**a**^	-4.911^**a**^	**I(1)**	-1.641	-1.516	**I(0)**	-4.785^**a**^	-3.474^**a**^	**I(1)**
**ENR**	-1.334	-1.309	**I(0)**	-4.798^**a**^	-4.825^**a**^	**I(1)**	-1.234	-1.139	**I(0)**	-4.982^**a**^	-4.521^**a**^	**I(1)**
**FDI**	-1.317	-1.321	**I(0)**	-4.872^**a**^	-4.799^**a**^	**I(1)**	-1.326	-1.218	**I(0)**	-5.012^**a**^	-4.764^**a**^	**I(1)**
**FIAD**	-1.337	-1.311	**I(0)**	-5.212^**a**^	-5.173^**a**^	**I(1)**	-1.621	-1.234	**I(0)**	-4.552^**a**^	-4.323^**a**^	**I(1)**
**CO**_**2**_	-1.223	-1.200	**I(0)**	-4.347^**a**^	-4.315^**a**^	**I(1)**	-1.261	-1.324	**I(0)**	-5.122^**a**^	-5.097^**a**^	**I(1)**
**TROP**	-1.246	-1.502	**I(0)**	-4.797^**a**^	-4.710^**a**^	**I(1)**	-1.321	-1.214	**I(0)**	-3.979^**a**^	-3.898^**a**^	**I(1)**
**SADC**												
**GDP**	-1.311	-1.571	**I(0)**	-4.822^**a**^	-5.075^**a**^	**I(1)**	-1.230	-1.327	**I(0)**	-4.766^**a**^	-4.752^**a**^	**I(1)**
**DFA**	-1.412	-1.103	**I(0)**	-5.244^**a**^	-4.957^**a**^	**I(1)**	-1.358	-1.261	**I(0)**	-5.059^**a**^	-4.778^**a**^	**I(1)**
**ENR**	-1.314	-1.216	**I(0)**	-4.714^**a**^	-4.809^**a**^	**I(1)**	-1.421	-1.303	**I(0)**	-5.012^**a**^	-4.709^**a**^	**I(1)**
**FDI**	-1.014	-1.125	**I(0)**	-4.692^**a**^	-5.024^**a**^	**I(1)**	-1.201	-1.701	**I(0)**	-4.775^**a**^	-4.879^**a**^	**I(1)**
**FIAD**	-1.135	-1.319	**I(0)**	-4.975^**a**^	-5.193^**a**^	**I(1)**	-1.332	-1.510	**I(0)**	-4.977^**a**^	-4.275^**x**^	**I(1)**
**CO**_**2**_	-1.117	-1.329	**I(0)**	-4.548^**a**^	-4.226^**a**^	**I(1)**	-1.137	-1.307	**I(0)**	-4.333^**a**^	-4.719^**x**^	**I(1)**
**TROP**	-1.216	-1.202	**I(0)**	-3.977^**a**^	-4.100^**a**^	**I(1)**	-1.406	-1.201	**I(0)**	-4.287^**a**^	-4.497^**x**^	**I(1)**
**ECOWAS**												
**GDP**	-1.528	-1.511	**I(0)**	-4.441^**a**^	-4.843^**a**^	**I(1)**	-1.343	-1.388	**I(0)**	-4.839^**a**^	4.972^**a**^	**I(1)**
**DFA**	-1.329	-1.235	**I(0)**	-4.387^**a**^	-4.902^**a**^	**I(1)**	-1.450	-1.350	**I(0)**	-5.205^**a**^	-5.015^**a**^	**I(1)**
**ENR**	-1.568	-1.392	**I(0)**	-5.347^**a**^	-5.203^**a**^	**I(1)**	-1.554	-1.538	**I(0)**	-4.989^**a**^	-4.977^**a**^	**I(1)**
**FDI**	-1.701	-1.433	**I(0)**	-5.037^**a**^	-5.139^**a**^	**I(1)**	-1.423	-1.374	**I(0)**	-5.109^**a**^	-5.216^**a**^	**I(1)**
**FIAD**	-1.211	-1.333	**I(0)**	-4.978^**a**^	-5.011^**a**^	**I(1)**	-1.747	-1.545	**I(0)**	-4.895^**a**^	-4.976^**a**^	**I(1)**
**CO**_**2**_	-1.313	-1.418	**I(0)**	-5.012^**a**^	-5.267^**a**^	**I(1)**	-1.732	-1.514	**I(0)**	-4.754^**a**^	-4.923^**x**^	**I(1)**
**TROP**	-1.376	-1.242	**I(0)**	-4.597^**a**^	-4.711^**a**^	**I(1)**	-1.620	-1.504	**I(0)**	-4.557^**x**^	-4.708^**x**^	**I(1)**

**Note:** Both the CADF and CIPS test the null hypothesis that the variables have unit root for each panel. "a, b, c" represent the level significance at 1%, 5%, 10% respectively.

### 4.5. Co-integration test results

The [44] co-integration was Used in evaluating the long-run relationship between the variables. In to the test statistics G_τ_, G_α_, G_τ_ P_α_, the alternate hypothesis was accepted at the significance levels. For comparison analysis, the [49] co-integration test was utilized. The results of the two co-integration outcomes are shown in Tables 8 and 9. The robust p-value, as seen in Table 8, which offers clear evidence for co-integration was the basis for decision-making. Seven statistics supporting the Pedroni panel cointegration test are shown in Table 9. Four test statistics accepted the alternate hypothesis (existence of co-integration).

**Table 8:** Westerlund bootstrap cointegration test.

Groupings	*G*_*τ*_		*G*_*α*_		*P*_*τ*_		*P*_*α*_	
	value	p-robust	value	p-robust	value	p-robust	value	p-robust
**AMU-EAC**	-3.737^**a**^	0.001	-3.771^**a**^	0.000	-4.889^**b**^	0.012	-3.545^**b**^	0.037
**CEN-SAD**	-4.631^**a**^	0.000	-7.718^**b**^	0.032	-8.443^**a**^	0.000	-10.212^**a**^	0.000
**COMESA**	-2.691^**b**^	0.030	-4.970^**b**^	0.042	-2.735^**a**^	0.000	-5.134^**b**^	0.043
**SADC**	-2.765^**b**^	0.041	-5.047^**a**^	0.000	-4.151^**a**^	0.000	-8.301^**b**^	0.020
**ECOWAS**	-5.691^**y**^	0.011	-6.537^**a**^	0.000	-3.732^**a**^	0.004	-5.340^**a**^	0.000

**Note**: a, b and c indicate 1%, 5% and 10% level of significance respectively. Probability of rejection of *H*_0_ are provided in (). Calculation of the P-values are based on one side of the normal distribution test.

**Table 9:** Pedroni cointegration test.

Groupings	Panel Statistics				Group Statistics		
	V-Stats	Rho-Stats	PP-Stats	ADF-Stats	Rho-Stats	PP-Stats	ADF-Stats
**AMU-EAC**	-5.410^**a**^ (0.000)	-3.711^a^ (0.021)	-6.712^**a**^ (0.000)	3.688 (0.312)	-5.235^**a**^ (0.000)	2.304 (0.330)	-2.135^**c**^ (0.072)
**CEN-SAD**	-2.786^**a**^ (0.031)	3.778 (0.340)	-4.598^**b**^ (0.010)	-7.223^**a**^ (0.000)	-2.872^**a**^ (0.041)	-4.660^**b**^ (0.031)	-2.346^**b**^ (0.037)
**COMESA**	1.353 (0.314)	-4.139^**a**^ (0.001)	-3.435^**b**^ (0.022)	-4.432^**a**^ (0.001)	-3.012^**b**^ (0.082)	2.562 (0.130)	-5.540^a^ (0.000)
**SADC**	-4.012^**a**^ (0.000)	2.077 (0.321)	-5.117^**b**^ (0.014)	-3.414^**b**^ (0.044)	-3.421^**a**^ (0.000)	-4.109^**a**^ (0.020)	2.217 (0.322)
**ECOWAS**	-5.330^**a**^ (0.000)	-3.830^**a**^ (0.042)	-2.843^**b**^ (0.032)	2.899 (0.320)	-3.465^**c**^ (0.051)	4.557 (0.501)	-7.695^**a**^ (0.000)

**Note**: ’a, b, c’ denote the acceptance of the alternative hypothesis at 1%, 5% and 10% level of significance. The test adopts the null hypothesis of no cointegration.

### 4.6. Parameter estimations results

Estimation of the long-run equilibrium relationship among the variables from the DSUR is presented in Table 10. Results from AMU-EAC show that a unit percentage increase in DFA, ENR, FDI, FIAD, and TROP turns to increase GDP by 0.033, 0.869, 0.091, 0.560, and 0.230 units, respectively at various level of significance. Likewise, in CEN-SAD, GDP has increased by 1.301, 1.035, 0.443, 2.035, 1.023, 0.241 units by a percentage gain in TROP, CO_2_, FIAD, FDI, ENR, and DFA, respectively. Whereas in the case of COMESA, a unit percent increase in DFA ENR FDI, CO_2_, and TROP correspondently indicated a significant increase of 0.501, 2.015, 0.773, 0.612, and units in GDP, respectively. A close look at SADC unveils that an increase of 1.205, 1.515, 2.033, 0.721 units in GDP was projected by a unit percent increase in DFA, ENR, FDI, and TROP, respectively. Lastly, in ECOWAS, a percentage increase in DFA, ENR, FDI, FIAD CO_2_, and TROP turns to significantly step up GDP by 1.175, 1.104, 0.859, 0.446, 0.434, 0.397 units, respectively. By comparing the elasticity of the determinants of GDP across various trading blocs groupings, the above variables are more beneficial to the actual GDP of CEN-SAD and ECOWAS, followed by AMU-EAC and COMESA, and SADC having the least significant effect of the employed variable. Thus it was concluded that the variables employed as determinants of per capita GDP were essential to GDP growth in Africa.

**Table 10:** Results from the DSUR estimation approach.

Grouping	Variables	Coefficient	Std.error	t-value	Prob.
**AMU-EAC**	**DFA**	0.033	0.067	7.69	0.031
	**ENR**	0.869	0.039	26.77	0.000
	**FDI**	0.091	0.050	50.11	0.009
	**FIAD**	0.560	0.037	32.26	0.022
	**CO**_**2**_	0.160	0.043	-3.21	0.231
	**TROP**	0.230	0.018	16.25	0.016
	**Constant**	-1.360	0.127	-21.72	0.002
		**R-Square**	0.932		0.000
		**F-Statistic**	78.92^**a**^		0.000
		**Observations**	260		
**CEN-SAD**	**DFA**	0.241	0.021	27.73	0.000
	**ENR**	1.023	0.046	18.29	0.020
	**FDI**	2.035	0.114	5.01	0.032
	**FIAD**	0.443	0.077	7.65	0.011
	**CO**_**2**_	1.035	0.123	5.57	0.042
	**TROP**	1.301	0.230	19.75	0.001
	**Constant**	-3.971	0.203	-14.02	0.013
		**R-Square**	0.911		0.000
		**F-Statistic**	45.01^**a**^		0.000
		**Observations**	434		
**COMESA**	**DFA**	0.501	0.044	22.05	0.002
	**ENR**	2.015	0.071	9.79	0.014
	**FDI**	0.773	0.098	15.18	0.009
	**FIAD**	0.060	0.027	-3.72	0.131
	**CO**_**2**_	0.612	0.023	11.96	0.031
	**TROP**	0.485	0.012	17.83	0.071
	**Constant**	-0.060	0.027	-3.72	0.052
		**R-Square**	0.877		0.000
		**F-Statistic**	67.92^**a**^		0.000
		**Observation**	406		
**SADC**	**DFA**	1.205	0.083	31.55	0.008
	**ENR**	1.515	0.075	15.78	0.000
	**FDI**	2.033	0.088	25.26	0.007
	**FIAD**	-0.214	2.378	0.22	0.171
	**CO**_**2**_	0.060	6.427	-0.72	0.352
	**TROP**	0.721	0.074	23.44	0.017
	**Constant**	1.010	0.027	43.72	0.003
		**R-Square**	0.951		0.000
		**F-Statistic**	54.72^**a**^		0.000
		**Observation**	348		
**ECOWAS**	**DFA**	1.175	0.191	23.09	0.004
	**ENR**	1.104	0.052	17.13	0.000
	**FDI**	0.859	0.072	9.36	0.022
	**FIAD**	0.446	0.027	13.32	0.015
	**CO**_**2**_	0.434	0.032	41.04	0.005
	**TROP**	0.397	0.546	20.17	0.016
	**Constant**	-1.120	0.027	-9.72	0.041
		**R-Square**	0.944		0.000
		**F-Statistic**	92.67^**a**^		0.000
		**Observation**	290		

**Note**: a, b and c indicate 1%, 5% and 10% level of significance respectively.

The R-square and the F-statistic values together affirm how well the model replicates the observed outcomes. This R-square is based on the proportion of the total variance of the outcomes explained by the model. After employing the DSUR for long-term estimation, the validity of the model is carefully tested. Therefore, the White heteroscedasticity test and Wooldridge series correlation test were used to evaluate the model’s effectiveness. The results shown in Table 11 indicate no definite heteroscedasticity and sequence correlation among the model residuals.

**Table 11:** Diagnostics test results.

	AMU-EAC		CENSAD		COMESA		SADC		ECOWAS	
	Value	Prob.	Value	Prob.	Value	Prob.	Value	Prob.	Value	Prob.
**WSC test**	34.233	0.412	42.324	0.511	37.451	0.158	51.103	0.431	74.302	0.521
**WH test**	14.411	0.356	10.543	0.361	12.124	0.506	32.704	0.317	32.111	0.341

**Note:** WSC test-Wooldridge Serial Correlation test, WH test-White Heteroscedasticity test.

### 4.7. Causality test

The evidence of the long-term connection between the variables suggests that at least one path must be causal ties, as estimated by the long term effects DSUR does not reveal the direction of the causal connection between the variables. Therefore, using a heterogeneous panel method, the variables’ causal liaisons are given in the next paragraph. Among the variables, a combination of the causal affiliations results is obtained in Tables 12–16, as some findings are consistent across national groupings. Others, by comparison, differ from one panel to another.

**Table 12:** D-H causality test for Arab Maghreb Union & East African Community.

	GDP	DFA	ENR	FDI	FIAD	CO_2_	TROP
GDP	**-**	**[4.747] 6.683a**	**[2.654] 2.862a**	**[5.440] 7.947a**	**[1.635] -0.635**	**[3.264] 3.976a**	**[6.926] 10.660a**
DFA	**[0.937] 1.854**	**-**	[3.483] 4.374a	[4.243] 5.763a	[3.586] 4.562a	[1.079] -0.014	[2.060] 1.776b
ENR	**[4.337] 5.457a**	[2.860] 2.860a	**-**	[3.494] 4.395a	[1.536] 0.820	[4.823] 6.820a	[3.936] 4.550a
FDI	**[5.062] 7.258a**	[3.583] [4.558]a	[1.326]a 0.437	**-**	[1.257] 0.310	[2.019] 1.702b	[0.132] 9.211
FIAD	**[2.419] 2.456b**	[4.022] 5.359a	[3.251] 3.952a	[2.235] 2.096b	**-**	[1.576] [0.894]	[1.169] [0.150]
CO_2_	**[4.034] 5.380a**	[4.707] 6.609a	[2.990] 3.474a	[8.045]a 12.7040a	[6.868] 10.555a	**-**	[3.301] [4.042]a
TROP	**[10.456] 17.104a**	[4.876] 6.917a	[8.400] 13.352a	[7.272] 11.291a	[3.222] 3.897a	[3.548] 4.493a	**-**

**Table 13:** D-H causality test for Community of Sahel-Saharan States.

	**GDP**	DFA	ENR	FDI	FIAD	CO_2_	TROP
GDP	**-**	**[4.519] 8.089a**	**[2.521] 3.380a**	**[7.791] 15.800a**	**[3.701] 6.161a**	**[1.579] 1.160**	**[2.269] 2.786a**
DFA	**[2.439] 3.186a**	-	[2.150] 2.506b	[1.530] 1.044	[3.290] 5.192a	[0.711] -0.884	[1.448] 0.852
ENR	**[3.751] 6.278a**	[3.808] 6.415a	-	[6.999] 13.934a	[1.895] 1.906	[3.703] 6.165a	[2.897] 4.267a
FDI	**[3.156] 4.877a**	[4.336]a 7.658a	[3.260] 5.123a	-	[2.187] 2.594a	[2.455] 3.226a	[2.238] 2.712a
FIAD	**[2.039] 2.245b**	[2.692]a 3.783a	[0.718] -0.869	[1.901] 1.920c	-	[0.759] -0.772	[2.491] 3.309a
CO_2_	**[5.001] 9.225a**	[6.274] 12.226a	[1.701] 1.447	[9.055] 18.779a	[4.157] 7.235a	-	[3.116] 4.783a
TROP	**[5.263] 9.844a**	[6.566] 12.911a	[4.828] 8.817a	8.384 17.198a	[2.939] 4.367a	[2.168] 2.548b	-

**Table 14:** D-H causality test for Common Market for Eastern and Southern Africa.

	GDP	DFA	ENR	FDI	FIAD	CO_2_	TROP
GDP	-	**[7.295] 12.376a**	**[7.152] 4.098a**	**[4.127] 5.107a**	**[3.129] 5.161a**	**[0.956] 1.096**	**[2.908] 4.346a**
DFA	**[3.910] 5.672a**	-	[3.332] 5.506b	[1.032] 0.592	[3.018] 5.256a	[0.144]-0.431	[1.872] 0.260
ENR	**[3.446]a 5.099a**	[5.902] 6.708a	-	[11.147] 7.046a	[1.573] 1.612	[3.307] 6.565a	[2.773] 4.762a
FDI	**[2.465]a 4.686a**	[5.663] 7.882a	[3.183] 4.038a	-	[2.783] 2.453a	[2.586] 3.622a	[2.805] 2.290a
FIAD	**[3.184] 4.275b**	[4.927] 2.440a	[0.519] -0.811	[1.169] 3.015c	-	[0.914] -0.260	[2.123] 3.959a
CO_2_	**[7.122] 5.714a**	[9.059] 5.462a	[1.411] 1.744	[3.559] 5.339a	[4.705] 7.559a	-	[3.656] 4.337a
TROP	**[4.098] 7.514a**	[7.059] 11.314a	[4.843] 8.690a	[4.432] 7.234a	[2.998] 4.720a	[2.848] 2.894b	-

**Table 15:** D-H causality test for Southern African Development Community.

	GDP	DFA	ENR	FDI	FIAD	CO_2_	TROP
GDP	**-**	**[3.192] 5.079**	**[3.871] 3.026a**	**[2.468] 4.023a**	**[5.857] 4.926a**	**[1.630] 1.317**	**[4.622] 2.221b**
DFA	**[3.733] 3.037b**	-	[1.103] 0.527	[1.232] -1.051	[5.347] 7.532a	[5.560] 5.899a	[2.084]c [3.188]c
ENR	**[4.519] 5.304a**	[3.199] [3.027b	-	[3.376] 8.141a	[3.348] 2.318b	[3.440] 4.217a	[3.350] 2.045b
FDI	**[3.117] 4.431a**	[3.184] 4.079a	[3.055] 5.190a	-	[2.033] [2.146]b	[2.322] 2.136a	[2.335] 3.420a
FIAD	**[3.230] 3.335a**	[3.118] 4.751a	[3.229] 2.367b	[2.470] 2.566c	-	[0.561] -0.112	[3.532] 5.025a
CO_2_	**[3.237] 4.014a**	5.227] 7.206a	[2.567] 3.207b	[3.402] 4.452a	[6.675] 8.436a	-	[2.324] 3.012a
TROP	**[5.425] 7.027a**	[5.040] 6.451a	[5.084] 3.239a	[3.180] 4.208a	[3.206] 4.345a	[1.447] 1.601	-

**Table 16:** D-H causality test for Economic Community of West African States.

	GDP	DFA	ENR	FDI	FIAD	CO_2_	TROP
GDP	**-**	**[4.510] 6.588a**	**[2.623] 2.956a**	**[3.613] 4.862a**	**[3.636] 4.906a**	**[1.700] 1.179**	**[2.415] 2.556b**
DFA	**[2.267] 2.272b**	-	[1.401] 0.604	[0.802] -0.546	[5.816] 9.100a	[0.669] -0.804	[1.970] 1.700c
ENR	**[4.214] 6.019a**	[3.714] 5.057a	-	[5.317] 8.141a	[2.292] 2.320b	[3.509] 4.661a	[3.127] 3.926a
FDI	**[4.506] 6.581a**	[3.702] 5.033a	[4.486] 6.541a	-	[2.610] 2.931b	[2.615] 2.940a	[2.650] 3.008a
FIAD	**[3.020] 3.681a**	[2.698] 3.100a	[1.298] 0.406	[1.528] 0.8500	-	[0.97551] [-0.21445]	[3.689] 5.008a
CO_2_	**[4.035] 5.674a**	[6.093] 9.633a	[1.412] 0.625	[4.991] 7.513a	[4.935] 7.404a	-	[2.738] 3.178a
TROP	**[6.986] 11.352a**	[5.455] 8.405a	[1.391] 0.586	[4.646] 6.850a	[3.524] 4.691a	[1.911] 1.587	-

Considering AMU-EAC, a two-way causation liaison was observed among (GDP-ENR), (GDP-FDI), (CO_2_-GDP), and (TROP-GDP). In comparison, a unidirectional causation effect was observed from GDP to DFA, while one unidirectional causation effect from FIAD to GDP was highlighted. In the case of CEN-SAD, a two-way causation effect was illustrated between (DFA-GDP), (ENR-GDP), (GDP-FDI), and (TROP-GDP). Whereas a unidirectional causation liaison was observed from FIAD from GDP, likewise from CO_2_ to GDP was highlighted. In the case of COMESA, it was highlighted that a two-way causation affiliation between (DFA-GDP), (ENR-GDP), and (TROP-GDP). Simultaneously, a unidirectional causation liaison was observed from FDI to GDP, from FIAD to GDP, and from CO_2_ to GDP. A close look at SADC unveiled a unidirectional causation effect from FIAD to GDP, from ENR to GDP, from DFA to GDP, and from CO_2_ to GDP. Likewise, a bilateral causation liaison was highlighted between (FDI-GDP) and (TROP-GDP). Lastly, considering ECOWAS, it was observed that a two-way causation liaison was observed between (ENR-GDP), (FDI-GDP), (CO_2_-GDP), and (TROP-GDP). Again, it was detected that a unidirectional causation effect was seen in DFA to GDP and from FIAD to GDP.

## 5. Discussion

This study aimed to unveil the relationship between foreign aid, energy consumption, economic growth, trade openness, and CO_2_ emissions in the presence of domestic financial assistance and foreign direct investment in major trading blocs in Africa. These blocs are the Arab Maghreb Union, East African Community, Community of Sahel-Saharan States, Common Market for Eastern and Southern Africa, Sothern African Development Community, and Economic Community of West African States. This study applied the Breusch-Pagan LM, Bias-corrected scaled LM, Pesaran scaled LM, Pesaran CD, Friedman test, and the Pesaran-Yamagata test of homogeneity unveiled the presence of cross-sectional dependence and heterogeneity of errors in the slope. The existence of heterogeneity and cross-sectional dependence entails any changes in any variable employed in a country that can affect other regional economies. This result is consistent with the done by [50]. They identified heterogeneity and cross-sectional dependence in their empirical study on the relationship between economic growth, population growth, and CO_2_ emissions in 128 countries. The panel unit root test (CIPS and CADF) usage pointed out that the series employed are stationary, I (1). Econometrically, it is vital to work with stationary variables to avoid spurious results during estimation. These results are in line with the study done by [36]. They ensured that the employed variables have no unit root before estimating the model when they explored the effect of CO_2_ emissions, globalization, and financial development in BRICS countries. So did [51] employed CADF and CIPS in assessing the stationarity of CO_2_ emissions and biomass consumption in BRICS countries. They concluded that the employed variables had unit roots at the level but after the first difference, the variables had no unit-roots. Regarding the long-run association, the Westerlund-Edgerton bootstrap cointegration employed revealed that there exists a long-run association among the employed variables. This result is in accordance with [52]’s work in assessing the nexus between CO_2_ emissions, economic growth, urbanization, and energy consumption in Belt and Road economies. Similarly to [53]’s work in OECD, where they unveiled the effects of research and development on economic growth. In this study, [53] identified the presence of long-term association among their selected variables.

On the contrary, the long-run association obtained in this study differs from the work done by [54]. In their study, [54] revealed that the long-term relationship does not exist between economic growth and electricity consumption in the 15 emerging countries.

Considering the explanatory variables’ long-run estimates on economic growth along with each group, the DSUR was employed. Except for COMESA and SADC, in the three other trading blocs, FIAD had a significant positive material impact on GDP. This outcome reveals that an increase in FIAD stimulates economic growth by financing educational infrastructure, health facilities, providing emergency relief, and possibly stabilizing these relevant economies, which could be affected by shocks. This outcome is in line with the work done by [11], who saw a positive effect of FIAD on GDP in south Asian countries. Among all trading blocs, FDI had a substantial effect on GDP, which indicates that FDI, which is mostly invested in the private sector and more connected to physical capital, turns to increase economic growth in all trading blocs. This result is in uniform with the work done by [6], who identified a statistically significance of FDI effect on GDP in income groupings in BRI countries. ENR was seen to have a significant effect on GDP in all trading blocs. This unveils that as much energy is consumed for economic purpose turns to heighten economic growth. This outcome is in line with the work done by [7] in ten energy-consuming counties. They pointed to a cause and effect of these two variables and stated that ENR had a significant effect on GDP. CO_2_ emissions in CEN-SAD, COMESA, and ECOWAS showed a vital material effect on GDP. This unfolds that an increase in emissions of CO_2_ turns to increase economic growth. Specifically, the results in turns point that these trading blocs release more CO_2_ due to the usage of non-renewable energy sources for their economic activities. This output is in line with the investigation done by [55] in 5 ASEAN countries. They unveiled the effects of economic growth on the environment. Thus they posit that heighten economic growth turns to decrease the quality of the environment. Considering TROP, it was highlighted that TROP has a significant impact on GDP in all trading blocs. This points out that a change in TROP correspondently causes an increase in GDP. This upshot is in resonance with the work done by [56] in their study of Asian emerging economies. They stated that increasing TROP turns to decrease the quality of the environment. Lastly, for DFA in all trading blocs, it turns out that it has a significant impact on GDP. This revealed that DFA could be observed as a country’s quest for economic growth. Hence it increases via domestic investment, literally turns to boost economic growth. These results align with the work done by [57] in BRI for the study on the impact of economic growth and financial development on environmental quality. In their study, they stated that DFA has a positive significant effect on GDP along the BRI route.

In terms of the causal liaison among the variables analyzed, GDP-ENR exhibited a two-way causal relationship in the AMU-EAC, CEN-SAD, COMESA, and ECOWAS. This result implies that GDP-ENR in these trading blocs is correlated, such that an increase in ENR triggers GDP to increase and vice versa. These yields are coherent with the work done by [58] during their work between economic growth, energy consumption, and CO_2_ emissions where a two-way causative effect was seen between ENR and GDP. Likewise, the two-way causal liaison between GDP-FDI seen in all trading blocs is supported by the work done by [10], who attested the fact increase in GDP increases FDI and vice versa. Considering the two-way causal relationship seen between GDP-CO_2_ exhibited in AMU-EAC and ECOWAS, it also indicates that these two variables are correlated such that a rise in one variable turns to affect the other. This effect is supported by the study done by [59], which observed a two-way causation between these variables in China. Again the two ways liaison seen between DFA-GDP in CEN-SAD and COMESA unveils that DFA is mutually related to GDP and vice versa. These upshots resonate with the study done by [9] in South-East Asia and South Asia, where they identified a two-way causation DFA-GDP. Lastly, the two-way causal effect between TROP-GDP seen in all trading blocs discloses that GDP-TROP is correlated, such that an increase in TROP triggers an increase in GDP and vice versa. This uncovers is in line with the work done by [2] in OECD countries, which stated that a two-way causative association exists between TROP-GDP. However, the unidirectional causation liaison seen from GDP to DFA in SADC and ECOWAS discloses that an increase in DFA turns to increase GDP in the long run. So does the one-way causal effect seen from FIAD to GDP in all trading blocs expose foreign aid turns to heighten GDP but not the vice-versa. This argument is supported by the work done by [10], in Sudan, which revealed that a unidirectional causal effect from FIAD to GDP was seen.

Likewise, the unidirectional causal effect from CO_2_ to GDP in CEN-SAD, COMESA, and SADC highlights the increase in CO_2_ correspondingly increases GDP and not vice versa. This effect can be substantiated with the work done by [60] in Azerbaijan during their investigation on the effect of economic growth on CO_2_ emissions. They also observed a one-way causal effect seen from CO_2_ to GDP

## 6. Conclusion and policy implications

The current study investigates the dynamic relationship between GDP, FIAD, CO_2_, TROP, ENR, DFA, and FDI in major trading blocs in Africa from 1990 to 2018. The study employed several techniques including panel unit root tests, panel co-integration test, panel long-run elasticity, and panel D-H causality approach to ascertain more authentic and reasonable results among the variables. A variety of conclusions were drawn accordingly.

Estimates from the DSUR affirmed that FIAD accelerates GDP growth in AMU-EAC, CEN-SAD, and ECOWAS, whereas FDI induces an increase in GDP in all trading blocs in Africa. Likewise, did that ENR heightens GDP in all trading blocks. The white heteroscedasticity test and the Wooldridge serial correlation test were employed to validate the model established in this study. To comprehend the causal relationship among the employed variables, it was discovered that a quick distinct relationship exists as FIAD has a unidirectional liaison to GDP in all trading blocs. ENR exhibited a bilateral causal effect with GDP in AMU-EAC, CEN-SAD, COMESA, and ECOWAS. While in SADC, a causal unidirectional impact from ENR to GDP was noticed. In ripple effect out the finding obtained, the following possible implications were deduced for this study;

1. The bidirectional causality between energy consumption and economic growth asserts that ensuring energy availability is necessary for achieving long-run economic growth. Since Africa is already suffering from extreme energy/electricity shortages, the government of various economic groupings in Africa should concentrate on building resources to ensure that the economy has sufficient energy supplies. Thence, in the presence of bidirectional causality, energy shortages have clear consequences for economic growth in the economy.
2. Economic growth and the CO_2_ emissions in the long run feedback each. This suggests that higher economic growth could occur at the cost of a cleaner environment, which will undermine the quality economic growth. Thus, to address this feedback, it is recommended that abatement of CO_2_ emissions activities be included in Africa’s central energy and environmental policy to abridge impairments related to CO_2_ emissions.
3. Because an increase in FIAD and FDI correspondingly raises economic growth, through an increase in energy consumption, stringent environmental legislation needs to be extended to both international and local companies to curtail CO_2_ emissions. Since multimillionaires companies turn to flee from developing countries where stringent environmental policies have been established to reduce pollution. Again, the awareness of spillover from FIAD and FDI companies to local businesses must be promoted.
4. In the long run, an increase in energy usage would also lead to high emissions of CO_2_ in the economy. Although energy resources are essential for higher economic growth, it as well contributes to environmental degradation. Therefore, the government should increase the share of renewable energy sources in the economy’s overall energy mix to increase energy availability.

The policy recommendations provided in this empirical study are precise and robust since the employed panel econometrics approaches are effective, taking into account cross-sectionally and heterogeneous dependent panel data.
